# Supplementary material for: Identification of Dioscorea opposite Thunb. CDPK gene family reveals that DoCDPK20 is related to heat resistance
Source: PeerJ. 2023 Sep 20;11:e16110. doi: 10.7717/peerj.16110 (PMC10517659; doi:10.7717/peerj.16110)
Supplement: Supplemental Information 2 — Note: GGATCC and GTCGAC are the restriction sites of Kpn I and BamH I [file peerj-11-16110-s002.docx]

| Primer name | Forward sequence | Reverse sequence | Annotation |
| --- | --- | --- | --- |
| DoCDPK20-CDS | GCGGTGGATGTTGAGGAC | GCCATTATCCGTGATGTG | Cloning of DoCDPK20 CDS and fused with pGM-T vector |
| DoCDPK20-Z | CGGGATCCATGCGCCACCTCCCGCCCCACCCTA | CGGGGTACCACTAAGCTCTTCAATTTCTATGTAT | Construction of pPZP221-DoCDPK20 plant expression vector |
| *DoCDPK1* | GAGGGGGCATTATAGCGAACG | CCAGGCTTGAAGAACACGGAGAG | qRT-PCR |
| *DoCDPK6* | GTCGTCTTCTTCACAGCCTCCT | ATCTCACGCCACACATCCTCAT | qRT-PCR |
| *DoCDPK7* | CCCTCCGCCAAGCCATCC | CTCGCGCCGCACATCCTC | qRT-PCR |
| *DoCDPK10* | TGGAGTTGAGAGATGGGTTCGT | GGTGTTGATGGCTTGGCTGG | qRT-PCR |
| *DoCDPK16* | CCAAACCTAAGCCCCTCTCCG | GCTCATCCTCACAAGCCTCCC | qRT-PCR |
| *DoCDPK17* | CCAAACCTAAGCCCCTCTCCGT | TGCTCATCCTCACAAGCCTCCC | qRT-PCR |
| *DoCDPK20* | TGGGCAGTCCTTATTACA | AACCAAATCCGAAGAGG | qRT-PCR |
| *DoCDPK22* | GCCCTGCTCATAGTCTGTTTTCG | TGTCCCTTGTTCTCCCCTTTCTT | qRT-PCR |
| 18S-rRNA | GATCGGAGTAATGATTAACAG | TTATGGTTGAGACTAGGACG | qRT-PCR actin gene |
| Reaction procedure | 95 °C, 30 s (pre-degradation), 1 cycle; 95 °C, 5 s (denaturation), 60 °C, 30 s, 40 cycles; 95 °C, 5 s (dissolution); 60 °C, 1 min, 95 °C, 15 s, 1 cycle | |  |

**Supplementary Table 1** The primers used in this study

Note: GGATCC and GTCGAC are the restriction sites of *Kpn* I and *Bam*H I
